# Characteristics of a lipase ArEstA with lytic activity against drug-resistant pathogen from a novel myxobacterium, *Archangium lipolyticum* sp. nov.

**DOI:** 10.3389/fmicb.2023.1320827

**Published:** 2024-01-04

**Authors:** Yang Zhou, Haixin Chen, Hongxia Jiang, Qing Yao, Honghui Zhu

**Affiliations:** ^1^Key Laboratory of Agricultural Microbiomics and Precision Application (MARA), Guangdong Provincial Key Laboratory of Microbial Culture Collection and Application, Key Laboratory of Agricultural Microbiome (MARA), State Key Laboratory of Applied Microbiology Southern China, Institute of Microbiology, Guangdong Academy of Sciences, Guangzhou, China; ^2^Guangdong Key Laboratory for Veterinary Pharmaceutics Development and Safety Evaluation, College of Veterinary Medicine, South China Agricultural University, Guangzhou, China; ^3^College of Horticulture, Guangdong Province Key Laboratory of Microbial Signals and Disease Control, Guangdong Engineering Research Center for Litchi, South China Agricultural University, Guangzhou, China

**Keywords:** *Archangium lipolyticum* CY-1, myxobacterial predation, secreted proteins, lipase, biological agent

## Abstract

Bacteriolytic myxobacteria are versatile micropredators and are proposed as potential biocontrol agents against diverse bacterial and fungal pathogens. Isolation of new myxobacteria species and exploration of effective predatory products are necessary for successful biocontrol of pathogens. In this study, a myxobacterium strain CY-1 was isolated from a soil sample of a pig farm using the *Escherichia coli* baiting method. Based on the morphological observation, physiological test, 16S rRNA gene sequence, and genomic data, strain CY-1 was identified as a novel species of the myxobacterial genus *Archangium*, for which the name *Archangium lipolyticum* sp. nov. was proposed. Subsequent predation tests indicated that the strain efficiently lysed drug-resistant pathogens, with a higher predatory activity against *E. coli* 64 than *Staphylococcus aureus* GDMCC 1.771 (MRSA). The lysis of extracellular proteins against ester-bond-containing substrates (tributyrin, tween 80, egg-yolk, and autoclaved drug-resistant pathogens) inspired the mining of secreted predatory products with lipolytic activity. Furthermore, a lipase ArEstA was identified from the genome of CY-1, and the heterologously expressed and purified enzyme showed bacteriolytic activity against Gram-negative bacteria *E. coli* 64 but not against Gram-positive MRSA, possibly due to different accessibility of enzyme to lipid substrates in different preys. Our research not only provided a novel myxobacterium species and a candidate enzyme for the development of new biocontrol agents but also reported an experimental basis for further study on different mechanisms of secreted predatory products in myxobacterial killing and degrading of Gram-negative and Gram-positive preys.

## Introduction

Myxobacteria are ubiquitous predatory bacteria with complicated multicellular morphogenesis and complex social behaviors. Compared to studies into their sophisticated social lifestyle, researches on cultured species and myxobacteria taxonomy are relatively lacking (Wang et al., 2022). Myxobacteria are presently classified as a phylum (Myxococcota) according to the genome analysis (Waite et al., 2020). Culture-independent methods have revealed a high diversity of myxobacteria in different environments (Wang et al., 2021; Zhang et al., 2023a). However, the number of currently cultured myxobacteria is still remarkably small because the usual dilution and plating techniques used for microorganism isolation are unlikely to reveal the presence of myxobacteria (Mohr, 2018). The baiting method for myxobacteria isolation and the repeated transfers for purification are time-consuming and difficult (Shimkets et al., 2006). Furthermore, the most recently identified new species of myxobacteria belong to the genera *Myxococcus* and *Corallococcus*, which are frequently isolated but not dominant in soil indicated by culture-independent methods (Chambers et al., 2020; Livingstone et al., 2020; Zhou et al., 2020; Petters et al., 2021), and relatively fewer members of less-studied myxobacteria species have been reported over the last decade (Ahearne et al., 2023). Therefore, more efforts are encouraged to explore and obtain the less-studied myxobacteria with an extended taxonomic range.

Another fascinating characteristic of most myxobacteria is their predation behavior, which involves killing other microbial species to consume their biomass. Their predation behavior on pathogens has wide application potential in biological control (Pérez et al., 2016). For example, the myxobacterium *Corallococcus* sp. EGB could inhibit the growth of different plant pathogenic fungi and showed good biological control against cucumber *Fusarium* wilt (Li et al., 2017). In fact, myxobacteria process certain features that make them effective biological agents, such as predation on other bacteria and fungi, formation of fruiting bodies with moderate resistance, and the ability to slide on solid surfaces for diffusion (Ye et al., 2020). These characteristics have made the application of myxobacteria in exploring new resources with excellent predation against various pathogens a hot research topic.

The predation process of myxobacteria includes using social gliding motility to search for prey and employing a series of synergetic predatory products to kill and decompose the prey cells. The ability of myxobacteria to kill prey is mostly attributed to the secretion of hydrolytic enzymes and secondary metabolites, which presumably lyse surrounding bacteria (Berleman and Kirby, 2009; Muñoz-Dorado et al., 2016; Pérez et al., 2016). Exploring predation factors and their mechanisms in predation is one of the important fields of myxobacteria predation. It has been reported that myxobacterial outer membrane vesicles loaded with various predation factors acted as predatory weapons (Berleman et al., 2014). Previous studies have indicated that myxobacteria produce diverse secondary metabolites involved in their predation (Xiao et al., 2011; Surup et al., 2014; Schulz et al., 2018). In contrast to secondary metabolites, the function of myxobacteria hydrolytic enzymes in predation has received less attention. The model myxobacterium *Myxococcus xanthus* produces a variety of hydrolytic enzymes such as proteases, peptidases, lipases, and glycoside hydrolases responsible for lysing prey biomass (Evans et al., 2012). Recently, Zhang et al. (2023b) reported three specialized glucanases from *Archangium* sp. AC19 that act as a cooperative consortium to efficiently decompose β-1,3-glucans of plant-pathogenic oomycetes *Phytophthora*.

Myxobacteria completely consume prey biomass with a group attack strategy to efficiently destroy the prey cell structures. Based on the prey structure, macromolecule components found in the cell wall, cytomembrane, and nucleus are targets of lytic weapons from myxobacteria. In fact, myxobacteria genomes encode numerous peptidases, glycoside hydrolases, polysaccharide lyases, and carbohydrate esterases (Schneiker et al., 2007; Zhou et al., 2021; Wang et al., 2022), which likely target the prey structure in the process of predation. For example, glycoside hydrolases from myxobacteria targeting peptidoglycan in Gram-positive bacteria or β-1,6-glucans in fungi cell walls have been characterized and shown to contribute to prey biomass disruption of predation (Li et al., 2019; Arend et al., 2021). However, it remains unknown whether myxobacteria lytic enzymes can target other components of prey cell structures and how to play roles in predation.

Here we reported a predatory myxobacterium strain CY-1 isolated from pig farm soil and the strain showed efficient lytic activity against drug-resistant pathogens. This promising biocontrol agent was identified as a potential new species and proposed as *Archangium lipolyticum* sp. nov. A secreted carbohydrate esterase (ArEstA) in the CY-1 genome was identified and expressed in *E. coli*. Furthermore, the enzyme activity, biochemical characteristics, and bacteriolytic activity against drug-resistant pathogens were evaluated.

## Materials and methods

### Isolation and purification of myxobacterium strain CY-1

Strain CY-1 was isolated from a soil sample collected at a pig farm (N 22°57′49″, E 115°19′23″) in the city of Shanwei, Guangdong Province, China. The *Escherichia coli* baiting method was used to isolate myxobacteria (Shimkets et al., 2006). The swarming predatory colonies or fruiting bodies were observed after incubation at 30°C for 7 days. Then the potential myxobacteria were purified by cutting the furthest swarm colony edge or fruiting body from soil samples and repeatedly transferring them onto fresh VY/2 agar (0.5% dried baker’s yeast, 0.1% CaCl_2_·2H_2_O, 1.5% agar). Strain CY-1 was finally purified and deposited at the Guangdong Microbial Culture Collection Center (GDMCC 1.3728^T^).

### Identification of strain CY-1

Morphological characterization was conducted to describe the isolated myxobacterium strain CY-1. Growth performance was tested on VY/2, MD1 (0.6% casein, 0.2% soluble starch, 0.2% MgSO_4_·7H_2_O, 0.04% CaCl_2_·2H_2_O), CTT (1% casein, 8 mM MgSO_4_, 10 mM Tris–HCl, 1 mM KH_2_PO_4_, pH 7.6), and CYE (1% casein, yeast extract 0.05%, 8 mM MgSO_4_, 10 mM Mops, pH 7.6) medium at 30°C. Morphogenesis was observed by stereomicroscope, phase contrast microscope (PCM), and transmission electron microscopy (TEM). The swarm and fruiting body were observed by stereomicroscope. For PCM, strain CY-1 was grown in MD1 broth for 3 days, centrifuged, washed, and responded with TPM buffer (10 mM Tris–HCl, 1 mM KH_2_PO_4_, 8 mM MgSO_4_, pH 7.6). The resuspended cells were pipped on the slide and observed using an Axioscope 5 PCM microscope with an oil lens (Carl Zeiss, Jena, Germany). For TEM, the centrifuged pellet was fixed with 3% glutaraldehyde, followed by a secondary fixative of 1% osmium tetroxide. Samples were resuspended in 100 μL 2% agar solution and left at 4°C overnight before sectioning. Sections were observed using a H7650 TEM microscope (Hitachi, Tokyo, Japan).

Utilization of peptone and carbohydrate resources was performed on minimal medium agar supplemented with a final concentration of 0.2% (w/v) as described by Garcia et al. (2014). The tested peptone and carbohydrate resources included casein, tryptone, yeast extract, beef extract, soy peptone, polypeptone, starch, galactose, lactose, fructose, glucose, sorbitol, mannitol, sucrose, maltose, trehalose, and xylose. Antibiotic sensitivity was tested on VY/2 agar supplemented with different antibiotics at a final concentration of 50 μg ml^−1^ at 30°C (Mohr et al., 2012). The tested antibiotics included ampicillin, apramycin, neomycin, bacitracin, gentamicin, tetracycline, erythromycin, chloramphenicol, nalidixic acid, streptomycin, rifampin, hygromycin, zeocin, and kanamycin.

Isolate CY-1 was initially identified by 16S rRNA gene sequencing using the primers set F27/1492R (Lane, 1991). Amplification and sequencing of the 16S rRNA gene were carried out as previously described (Lachnik et al., 2002). The 16S rRNA gene sequence was compared with the EzTaxon database (Yoon et al., 2017a) to identify the most similar species.

The genome-based method was further used to identify strain CY-1, which has been proposed as the new standard for sequence-based taxonomic assignment (Richter and Rosselló-Móra, 2009). The genome of strain CY-1 was sequenced and used for genome-based identification. Briefly, a swarm of strain CY-1 was inoculated in MD1 liquid medium and incubated in a shake flask at 30°C for 3 days. Genomic DNA was extracted from spin-down biomass and sequenced by Majorbio (Shanghai, China) on the Illumina Hiseq 4000 platform using PE250. After quality control, the high-quality reads were assembled to contigs using SPAdes 3.13.1 (Bankevich et al., 2012). Then the contigs file was input into CheckM to estimate the genome completeness and degree of contamination (Parks et al., 2015). Prodigal (Hyatt et al., 2010) and Prokka (Seemann, 2014) were used for gene prediction and genome annotation, respectively. Additionally, the whole-genome phylogeny of strain CY-1 and related species was generated using the UBCG pipeline 3.0 (Na et al., 2018). The genomic taxonomy based on overall genome-relatedness indices digital DNA–DNA hybridization (dDDH) and average nucleotide identity (ANI) of strain CY-1 and related species were also used to identify the isolate. The dDDH values were calculated using the Genome-to-Genome Distance Calculator GGDC 3.0 (Meier-Kolthoff et al., 2022), and the ANI values were estimated using the online ANI calculator^1^ (Yoon et al., 2017b).

### Predation of drug-resistant bacteria by CY-1

CY-1 was incubated on VY/2 medium at 30°C for 3 days. The drug-resistant pathogens (Methicillin-resistant *Staphylococcus aureus* GDMCC 1.771, extended-spectrum β-lactamase CTX-M-27-producing *Escherichia coli* 64, Zhao et al., 2021) were inoculated in nutrient broth and shaken at 180 rpm and 30°C, respectively. The overnight shaken cultures were collected by centrifugation and resuspended in TPM buffer to the optical density of OD_600_ = 5. Then, 200 μL of pathogen suspension was spotted in the center of the TPM solid medium. When the pathogen bacteria were air-dried to form lawns, a 5 mm diameter plug strain of CY-1 was inoculated on the center of the pathogen colony. Three biological replicates were set up for each treatment and incubated at 30°C for 4 days. The zones of predation were observed after 2 days (early stage) and 4 days (late stage) of co-culture using a stereomicroscope and the imaging software (Olympus SZX10, Olympus Corporation, Tokyo, Japan), and the viable prey cells from the TPM plates were scraped, washed, and counted on nutrient broth solid plates via serial dilution. The colony-forming units (CFU) were obtained after incubating the plates at 30°C overnight.

### Lysis of biomacromolecules by CY-1

The WAT agar (0.1% MgSO_4_·2H_2_O, 0.02% K_2_HPO_4_, 1.5% agar, pH 7.6) added with 0.5% substrate was used to determine the lytic actions of CY-1 toward starch, chitin, tween, cellulose, and skim milk. The strain CY-1 was inoculated on the substrate agar, and the lytic actions were recorded as described (Shimkets et al., 2006). Considering that hydrolytic enzymes involved in outside prey-lysis are one of the mechanisms of myxobacteria predation, the lytic action of CY-1 liquid culture supernatant was also evaluated. Briefly, cell-free VY/2 culture supernatant was obtained by centrifugation of 250 mL culture 2 times for 15 min at 8,000 rpm and sequential filtration using 0.45-μm and 0.22-μm pore size filters. The filtered supernatant was concentrated by ultrafiltration with the molecular cut-off of 10 kDa. Considering the lytic action of CY-1 on Tween 80, we also tested the lysis of CY-1 extracellular proteins on ester-bond substrates. The filter paper disk diffusion method on TPM agar mixed with the autoclaved drug-resistant pathogen suspension (OD_600_ = 1) or substrate (0.5% tween, egg-yolk, tributyrin) was used to evaluate the lytic activity of crude extracellular proteins, respectively. The filter paper disks (5 mm diameter) were loaded with 20 μL of concentrated supernatant and placed on the TPM agar. Then the agar plates were incubated at 30°C and the transparent zone formed along the filter paper disks was observed and noted. Three replicates were set for each treatment. Heated extracellular proteins were also included as controls.

### Cloning, expression, and purification of the potential predatory factor ArEstA

We further focused on the alpha/beta hydrolases superfamily, which is one of the largest groups with diverse catalytic functions, such as hydrolysis of ester and peptide bonds. We searched for alpha/beta hydrolase domain protein in deduced amino acid sequences from the CY-1 genome based on the HMMER search using Pfam hidden Markov models (Eddy, 2009). An extracellular alpha-beta hydrolase (ArEstA) annotated to esterase in the CY-1 genome was identified in this study. As one of the potential predatory factors, the bacteriolytic activity of ArEstA against drug-resistant pathogens was evaluated.

The sequence of ArEstA was analyzed by the SignalP web server.^2^ The gene of ArEstA without signal peptide was cloned into the pET28a(+) (Invitrogen) expression vector at EcoRI and HindIII restriction sites. The obtained plasmid was transformed in *E. coli* BL21(DE3) electrocompetent cells. *E. coli* BL21(DE3) carrying the recombinant plasmid was shaken overnight at 37°C in LB broth with 100 ng μl^−1^ kanamycin. The culture was then inoculated (2%) into fresh LB broth with kanamycin and grown at 37°C until OD_600_ reached 0.6. Then the IPTG was added at a final concentration of 0.1 mM and the culture was incubated overnight at 16°C, 180 rpm for the induction of the expression of ArEstA. The cells were harvested and washed twice in ice-cold lysis buffer (137 mM NaCl, 2.7 mM KCl, 10 mM Na_2_HPO_4_, 1.8 mM KH_2_PO_4_, pH 8.0) and resuspended in the same buffer. After ultrasonic disruption, lysates were centrifuged to remove cell debris. The supernatant was passed through a 0.45 μm filter and then applied to metal-chelating Ni-NTA affinity chromatography (P2233, Beyotime, China) equilibrated with lysis buffer. After being washed in washing buffer (50 mM KH_2_PO_4_, 300 mM NaCl, 2 mM imidazole, pH 8.0), the Ni-NTA bound ArEstA was eluted using elution buffer (50 mM KH_2_PO_4_, 300 mM NaCl, 150 mM imidazole, pH 8.0) and dialyzed against lysis buffer. Sodium dodecyl sulfate-polyacrylamide gel electrophoresis (12%) was performed to verify molecular weight and purity.

### Characterization of ArEstA

Purified ArEstA was subjected to several biochemical assays including optimum temperature and pH, effects of metal ions on enzyme stability, and substrate specificity. The data were expressed as an average of the results from triplicate assays. Esterase activity was measured spectrophotometrically according to the method described (Cheng et al., 2011), using *p*-nitrophenyl (*p*NP) butyrate (C4) or *p*NP esters of other fatty acids (C2, C8, C12, and C16) as the substrates. The purified enzyme was incubated with a 200 μL reaction mixture containing 10 mM substrates, 1% ethanol, and 50 mM Tris–HCl (pH 8.0). The amount of *p*-nitrophenol released from *p*NP esters was monitored at 410 nm for 10 min by a Multiskan GO spectrophotometer. The activity was expressed as the release of 1 μmol of *p*-nitrophenol per minute. Protein concentration was determined by Bradford’s method using bovine serum albumin as standard. The filter paper disk diffusion method described above was also used to test the lytic ability of ArEstA toward ester-bond substrates (0.5% tween, egg-yolk, and tributyrin). The optimal pH for enzyme activity was determined by measuring the activity at pH ranging from 4.0 to 10.0 (50 mM citric-phosphate buffer for pH 4.0–6.0, 50 mM phosphate buffer for pH 6.5–7.5, 50 mM Tris–HCl buffer for pH 8.0–10.0). The effect of temperature on the activity of ArEstA was examined in 50 mM Tris–HCl buffer (pH 8.5) ranging from 20°C to 65°C by measuring the activity as the above method. The effect of metal ions (5 mM Mg^2+^, Mn^2+^, Cu^2+^, Zn^2+^, Fe^2+^, Fe^3+^, Co^2+^, Ni^2+^, Ba^2+^, Ca^2+^, K^+^, and Na^+^) on enzymatic activity was assessed in 50 mM Tris–HCl buffer (pH 8.5) at 45°C.

### Lytic action of ArEstA

The lytic action of ArEstA on the pathogen was performed by the filter paper disk diffusion method described above with some modification. The fresh drug-resistant pathogen suspension was used and the incubation time lasted for 48 h. Furthermore, the lytic action of ArEstA on the pathogen was also determined by the viable plate counting method. The pathogens at the mid-logarithmic phase were collected, washed, and resuspended in 50 mM phosphate buffer. The 200 μL reaction mixture contained ArEstA (0.5 mg/mL) and resuspended pathogen (final OD_600_ = 0.1). Controls were incubated in the absence of ArEstA. The mixture was incubated at 37°C for 2 h. Then the mixture was serially diluted and spread on nutrient agar plates. The CFU was obtained after incubating the agar plates at 30°C overnight. All assays were performed in triplicate and the results are the means of three independent experiments. Based on the results of the lytic activity of ArEstA against pathogens, the same mixture from *E. coli* 64 was obtained and used to indicate the lytic action using an H7650 TEM microscope (Hitachi, Tokyo, Japan).

### Sequence analysis and homology modeling

The amino acid sequences of ArEstA and related esterases were aligned using the Clustal Omega web server^3^ (Sievers and Higgins, 2018). The homology model structure was created by the SWISS-MODEL web server^4^ (Waterhouse et al., 2018). Verify_3D (Luthy et al., 1992) was used to check the residue profiles of the 3D models obtained. PROCHECK (Laskowski et al., 1996) analysis was performed to assess the stereochemical qualities of the 3D models. Pymol software (DeLano and Bromberg, 2004) was used to view the structure and generate figures.

## Results

### Isolation and identification of strain CY-1

**Figure 1:**
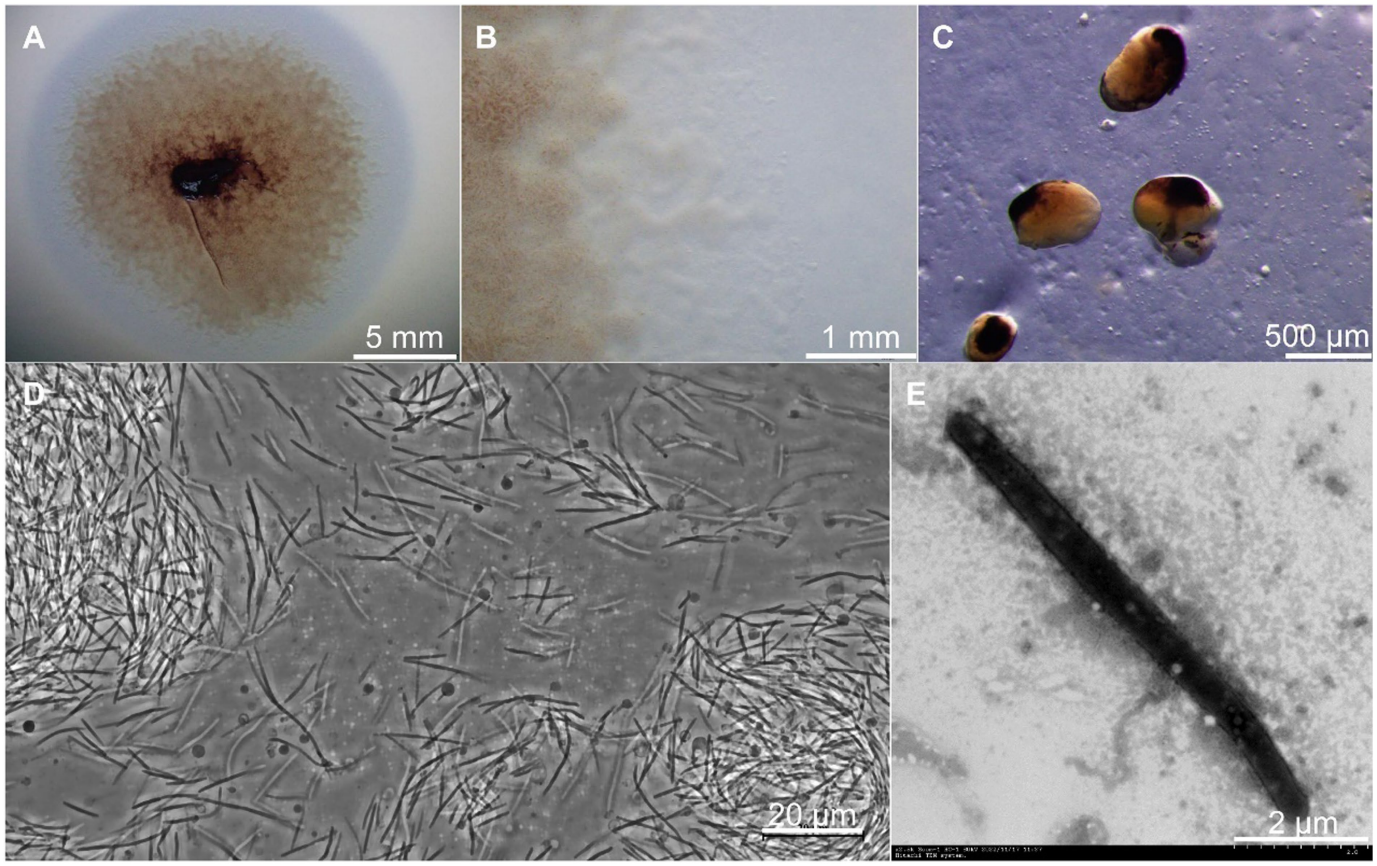
Growth and morphology of strain CY-1. **(A)** The spreading swarm colony of CY-1 on VY/2 agar after the 3-day cultivation. **(B)** Colony edge on VY/2 agar with defined veins and flared edges. **(C)** The dark brown and bean-shaped fruiting bodies formed on VY/2 agar. The long slender, needle-shaped rods of vegetative cells were observed using a phase contrast microscope **(D)** and transmission electron microscopy **(E)**. The scale bar was noted at the bottom right of each panel.

Strain CY-1 was isolated from pig farm soil by the *E. coli* baiting method for myxobacteria isolation. The colony expanded to form a swarm on VY/2, MD1, and CTT agar and formed fruiting bodies on VY/2 agar after starvation. A swarm of strain CY-1 showed yellow brown to purple color on VY/2 agar with a film-like appearance (Figure 1A) and significant veins and flares were observed on the periphery of the swarm (Figure 1B). The sessile fruiting bodies appeared on VY/2 agar for the 3-day cultivation and matured to dark brown and bean-shaped for 5 days (Figure 1C). The vegetative cells collected from MD1 agar were long slender, needle-shaped rods with rounded poles measuring 0.5 ~ 0.6 × 5.0 ~ 12.0 μm with a mean length of 6 ~ 8 μm indicated by phase contrast microscope and TEM (Figures 1D,E). Testing for growth at different temperatures (buffered VY/2 with pH 7.5) demonstrated that CY-1 grew at a temperature of 25 ~ 40°C. The optimum pH of CY-1 was 7.5. Substrate lytic action tests indicated that skim milk, starch, and Tween 80 were efficiently lysed by CY-1. This strain utilized all the tested peptone, starch, galactose, lactose, galactose, mannitol, sucrose, maltose, and lactose. The novel isolate was resistant to apramycin, hygromycin, gentamicin, ampicillin, kanamycin, and bacitracin, but was sensitive to zeocin, chloramphenicol, tetracycline, streptomycin, rifampicin, nalidixic acid, neomycin, and erythromycin.

**Figure 2:**
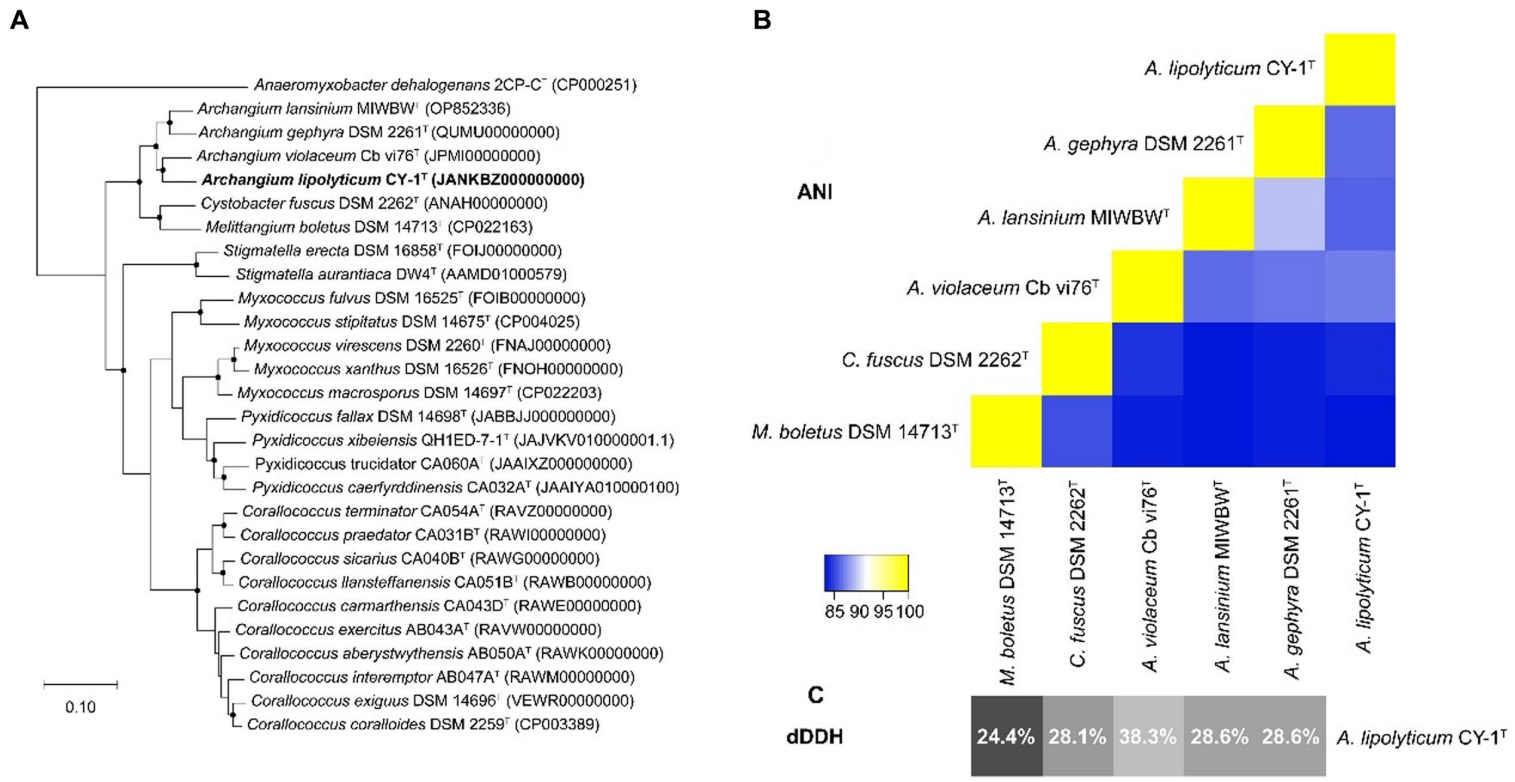
Genome-based identification of strain CY-1. **(A)** Phylogenetic tree based on genome sequence of strain CY-1 and the closely related type strains. Dots on branches indicate the bootstrap value >80%. *Anaeromyxobacter dehalogenans* 2CP-C^T^ was used as an outgroup to root the tree. The strain CY-1 was noted in bold font. **(B)** Heatmap generated from ANI values between CY-1 and the related species estimated using the online ANI calculator (https://www.ezbiocloud.net/tools/ani). **(C)** Grey-scale map generated from dDDH values between CY-1 and the related species calculated using the Genome-to-Genome Distance Calculator GGDC 3.0.

The 16S rRNA gene of CY-1 showed closest similarity to *A. violaceum* Cb vi61^T^ (99.03%), *A. minus* Cb m2^T^ (98.61%), *A. disciforme* CMU 1^T^ (97.71%), and *A. gephyra* DSM 2261^T^ (97.64%), suggesting the relatedness to members of genus *Archangium*. To further identify strain CY-1, the draft genome of strain CY-1 was obtained. The draft genome of strain CY-1 was 12.6 M with a 68.5% GC content spread over 75 contigs, with an N50 of 332.6 kb and an L50 of 12. The Prokka-based annotation of the draft genome identified 10,017 protein-coding genes and 75 non-coding genes. The genome-based phylogenomic tree indicated that CY-1 showed the highest similarity to *Archangium*, and represented a new clade located in the genus *Archangium* (Figure 2A). *In silico* genome-to-genome comparisons unambiguously showed that strain CY-1 possessed 24.4 ~ 38.3% dDDH and 83.23 ~ 86.88% ANI values with other species of *Archangium*, which were below the cut-off values for novel species (<70% dDDH, <95% ANI) (Goris et al., 2007; Meier-Kolthoff et al., 2013). CY-1 showed the highest dDDH and ANI values to *A. violaceum* Cb vi61^T^ (Figures 2B,C). Based on genomic and phylogenetic differences, we proposed that the candidate strain described here represented a novel species of *Archangium*.

### Predation and lytic action of strain CY-1 on drug-resistant pathogens

**Figure 3:**
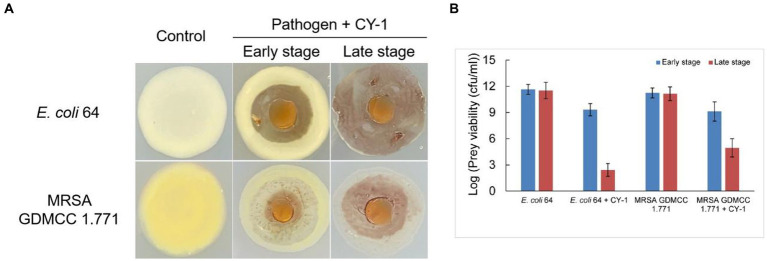
The predation behavior **(A)** and predatory activity [**(B)**, variable prey cell numbers after 2 days (early stage) and 4 days (late stage) co-incubation] of CY-1 against drug-resistant pathogens.

Strain CY-1 was able to prey on the tested drug-resistant bacteria in the plate assay, resulting in the lytic zone of the lawn of pathogen colonies (Figure 3A). After 4 days of co-culture, the pathogen colonies were lysed and overlaid by the growing cells of CY-1. The social predatory behaviors against different pathogens seemed to be different. Strain CY-1 extended fast with a thin and transparent swarm against MRSA but finally accumulated less biomass than the Gram-negative bacteria *E. coli* 64 (Figure 3A). The cell number of viable *E. coli* 64 and MRSA decreased after 2-day co-culture (early stage) and drastically decreased after 4-day co-culture (late stage). The cell number of viable *E. coli* 64 seemed less than that of MRSA but the variation was not significant (Figure 3B).

Then we focused on the secreted predatory products of strain CY-1 involved in the lysis of drug-resistant bacteria. In addition to the lysis of strain CY-1 against drug-resistant pathogens, the concentrated extracellular proteins collected from the fermentation supernatant of CY-1 also showed lytic activity toward autoclaved pathogens. Furthermore, the concentrated extracellular proteins of CY-1 lysed tween 80, egg-yolk, and tributyrin, suggesting that predatory product(s) in the crude proteins likely break down ester bond substrates of prey cells during predation (Table 1).

**Table 1:** The lysis of extracellular proteins on autoclaved drug-resistant bacteria and ester bond substrates.

Substrate	Extracellular proteins	Heated extracellular proteins
Autoclaved *E. coli* 64	++	–
Autoclaved MRSA GDMCC 1.771	+	–
Tween 80	+	–
Egg-yolk	+	–
Tributyrin	+	–

++, strong lysis; +, weak lysis; −, no lysis.

### Heterologous expression and biochemical characterization of ArEstA from strain CY-1

**Figure 4:**
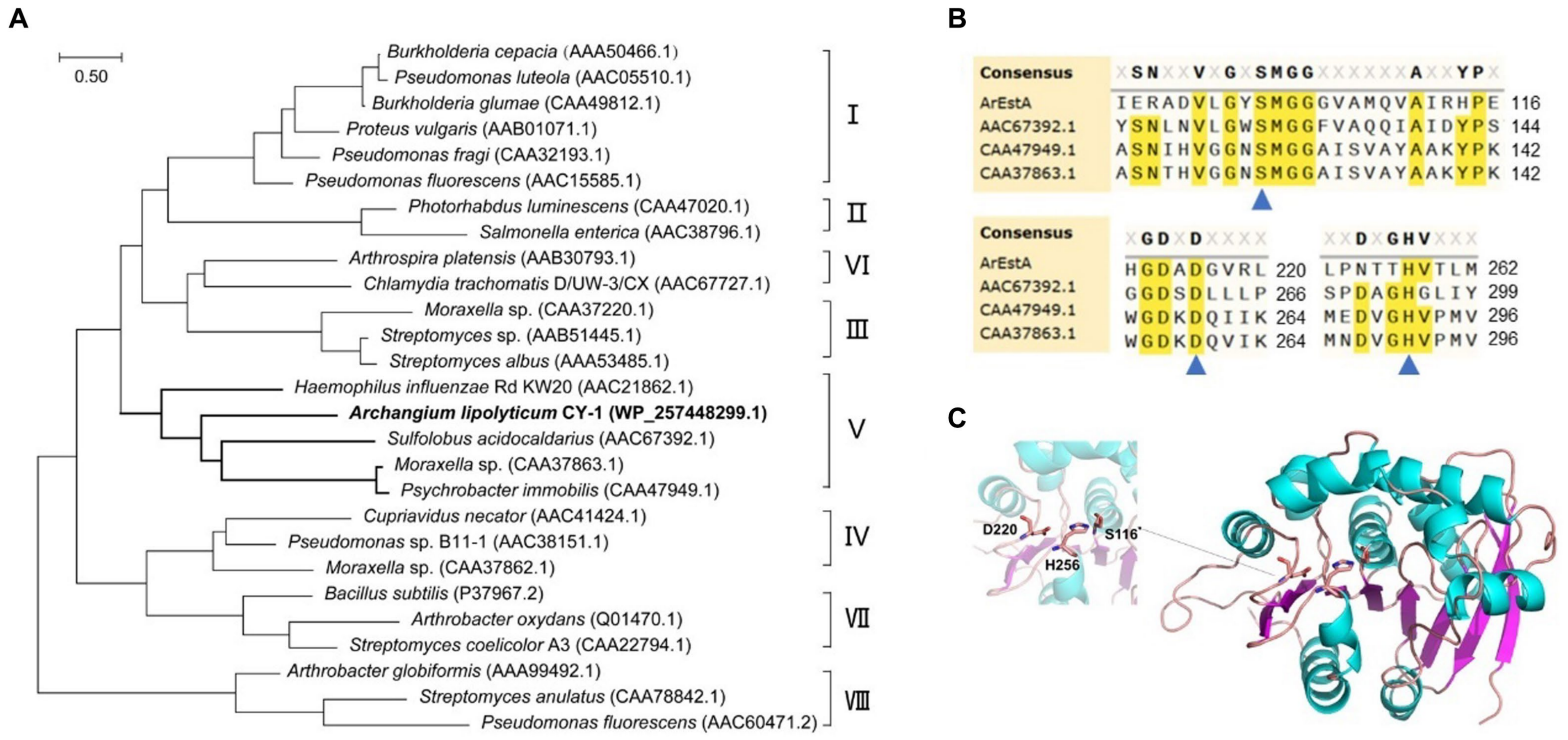
Sequence and structure analysis of ArEstA. **(A)** Phylogeny of ArEstA and the characterized lipolytic enzymes. ArEstA of myxobacterium *A. lipolyticum* CY-1 was noted in bold font. **(B)** Multiple sequence alignment of ArEstA and representative sequences from family V. The catalytic residues were indicated by blue triangles. **(C)** The model structure of ArEstA. The predicted catalytic residues of ArEstA were shown in sticks and labeled.

To explore the secreted predatory product(s) in the crude proteins of strain CY-1 with the ester-bond lytic activity and potential antibacterial activity, we analyzed its genome-encoded proteins that might act on ester or lipid. Because the superfamily of alpha/beta hydrolases is responsible for the hydrolysis of ester bonds, this conserved domain was explored along the genome of CY-1. The protein WP_257448299.1 assigned as ArEstA was chosen for further exploration as a putative extracellular esterase. The phylogenetic relationship between ArEstA and lipolytic enzymes representing the families proposed by Arpigny and Jaeger (1999) indicated that ArEstA was related to lipolytic enzymes from *Sulfolobus acidocaldarius* (accession number AAC67392.1), *Moraxella* sp. (accession number CAA37863.1) and *Psychrobacter immobilis* (accession number CAA47949.1), sharing 20, 18, and 17% amino acid sequence identity with ArEstA, respectively. According to Arpigny and Jaeger (1999), these enzymes belong to family V of bacterial lipolytic enzymes (Figure 4A). Analysis of these ArEstA proteins by multiple sequence alignment and the homology model based on the AlphaFold DB model of carboxylesterase (A0A3E0AS93_9BACT) revealed that both the pentapeptide motif Gly114-Tyr115-Ser116-Met117-Gly118 and the catalytic triad Ser116-His256-Asp220 were conserved in ArEstA. The catalytic nucleophile Ser is located in the central portion of the conserved motif G-X-S-M-G-G (Figures 4B,C), which is a sequence pattern of family V (Arpigny and Jaeger, 1999). Based on the sequence and structure analysis, we speculated that ArEstA might represent a new member in family V of lipolytic enzymes.

**Figure 5:**
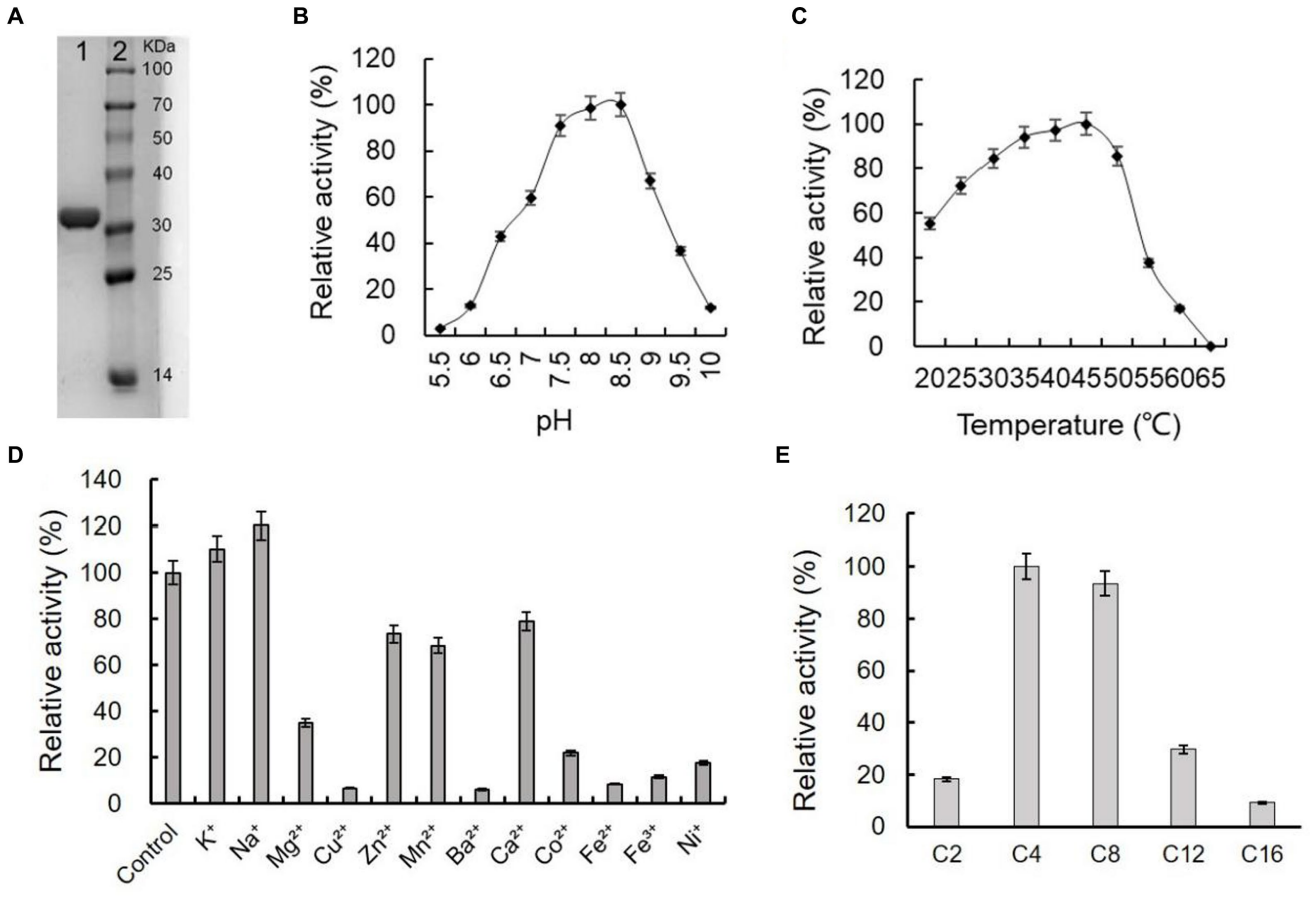
Purification and biochemical characterization of ArEstA. **(A)** SDS-PAGE analysis of the purified ArEstA. Lane 1, the purified protein of ArEstA protein, Lane 2, molecular weight marker. The effect of pH **(B)**, temperature **(C)**, and metal ions **(D)** on the activity of ArEstA. **(E)** Enzyme activities of ArEstA on different substrates.

The gene of ArEstA without signal peptide was cloned into the pET-28a vector and transformed into *E. coli* BL21(DE3) to express the enzyme protein. The purified ArEstA was a highly soluble protein of approximately 32 kDa (Figure 5A), consistent with the expected molecular weight. ArEstA showed lytic activity against *p*NP-C4 and *p*NP-C16 with a specific activity of 9.59 U/mg and 2.05 U/mg, respectively. ArEstA also showed hydrolysis toward tween 80, egg-yolk, and tributyrin.

Effects of pH on the enzymic activity of ArEstA were measured in a pH range from 4.0 to 10.0 with *p*NP butyrate as the substrate. The protein exhibited the maximum activity at pH 7.5–8.5 and the activity was almost completely lost at pH 5.5 (Figure 5B). The results suggested that ArEstA showed the optimum activity in alkalescent pH conditions. The protein showed the maximum activity at 35–45°C and the relativity still reached more than 70% at the temperature range from 30°C to 50°C. However, the activity was completely lost at 65°C (Figure 5C). These data indicated that ArEstA was a mesothermal enzyme. The enzyme activity was enhanced by the presence of 5 mM Na^+^ to 1.2-fold. The enzyme activity was strongly inhibited by Cu^2+^, Ba^2+^, Fe^2+^, Fe^3+^, and Ni^+^, and little or not affected by the presence of K^+^, Zn^2+^, Mn^2+^, and Ca^2+^ (Figure 5D).

The substrate specificity of ArEstA against *p*NP fatty acyl esters with various lengths of the acyl chain was assayed at pH 8.5 and 30°C. The ArEstA exhibited high activity against short-chain fatty acids of *p*NP butyrate (C4) and *p*NP octanoate (C8) except *p*NP acetate (C2). The activity significantly decreased to 29.7 and 9.1% for *p*NP dodecanoate (C12) and *p*NP palmitate (C16), respectively (Figure 5E).

### Lytic action of ArEstA against drug-resistant pathogen

**Figure 6:**
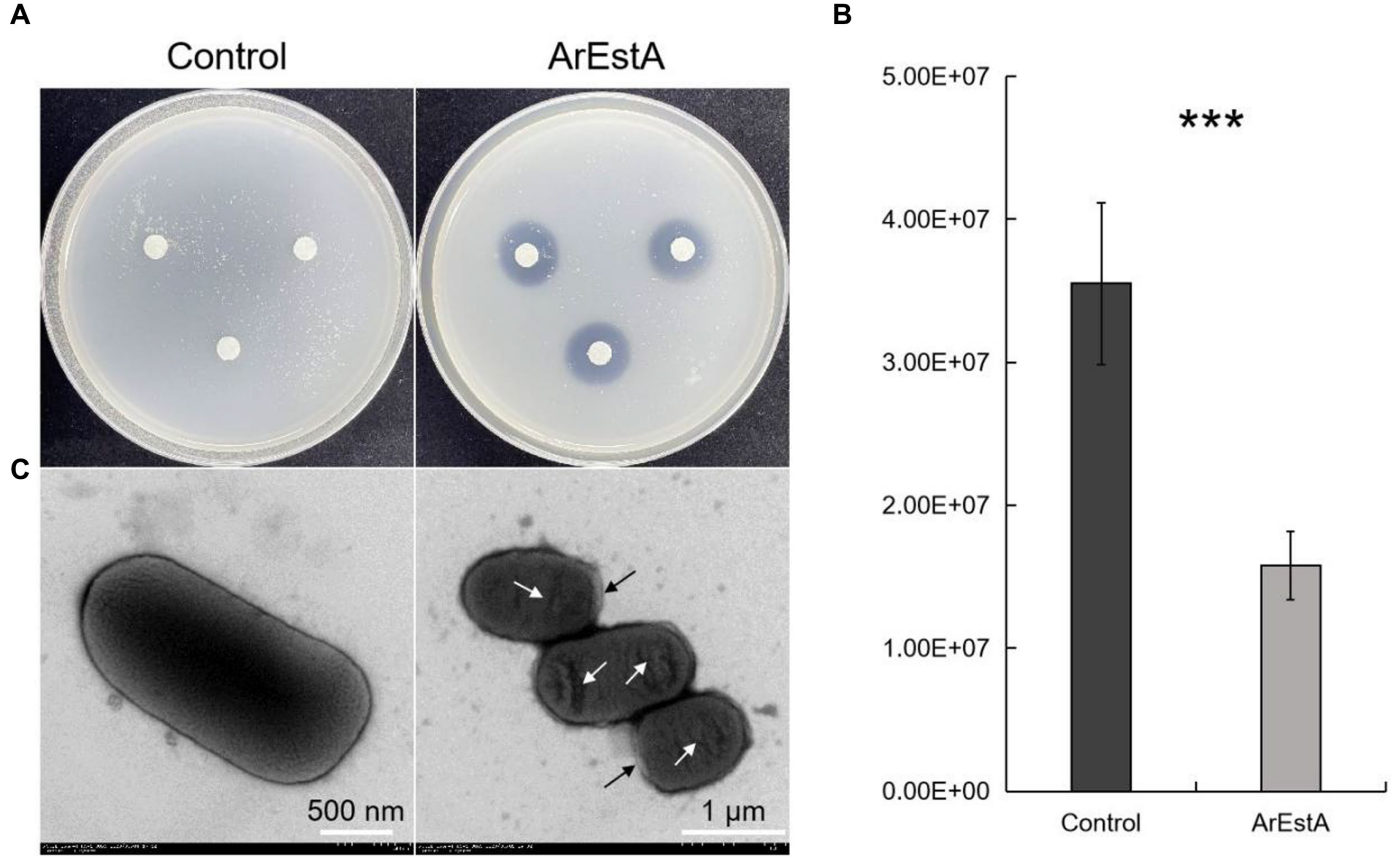
Bacteriolytic activity of ArEstA against *E. coli*. **(A)** Lytic activity of ArEstA on *E. coli* using the filter paper disk diffusion method. Plate count assays **(B)** and transmission electron microscopy observation **(C)** for the antimicrobial activity of ArEstA on *E. coli*. The black and white arrow indicated disruption and wrinkle of the cell envelope of *E. coli* treated with ArEstA, respectively. ***p < 0.001.

Since the predatory product(s) in the concentrated extracellular proteins of CY-1 can break down ester bond of prey cell component during predation, we explored whether the lipolytic enzyme ArEstA show lytic activity against drug-resistant pathogen like the predator CY-1. As shown in Figure 6, significant bacteriolysis was only observed on the *E. coli* plate treated with ArEstA, which to some extent was consistent with the strong lytic activity of extracellular proteins against *E. coli*. Then the antibacterial activity of ArEstA was further tested using viable plate counting assays. The number of viable *E. coli* cells treated with 0.5 mg mL^−1^ ArEstA was significantly decreased compared with control (Figure 6B), but ArEstA did not show antibacterial activity against MRSA. Furthermore, TEM showed the disruption and wrinkle of the cell envelope of *E. coli* treated with ArEstA (Figure 6C).

## Discussion

Myxobacteria have been proposed as potential biocontrol agents and have shown effective biocontrol activity against bacterial and fungal pathogens (Pérez et al., 2016). Isolation of new myxobacteria species and exploration of effective predatory products are necessary for successful pathogen biocontrol. We reported a new myxobacterium isolation of the *Archangium* and provided a candidate hydrolase showing bacteriolytic activity against Gram-negative drug-resistant bacteria in this study. The genus *Archangium* belongs to the less well-studied group Archangiaceae of Myxococcales, which is sibling to the largest group Myxococcaceae of the recently classified phylum Myxococcota (Waite et al., 2020). Traditionally, colony and fruiting body morphology are important indicators for myxobacteria taxonomy; however, the phase variation in myxobacteria can result in colonies with differences in pigment, texture, and swarming capabilities, which may complicate myxobacteria morphological taxonomy (Furusawa et al., 2011). Recently, it has been increasingly appreciated that limitations of 16S rRNA phylogenetic-based taxonomic assignment can be overcome by considering more genes, ultimately considering every gene of the bacterial genome (Livingstone et al., 2020; Liu et al., 2021). The relatively high 16S rRNA gene similarity of CY-1 to its closest type strain (99.03%) suggested that more genes were needed for accurate identification of the isolate. In this study, the genome-based phylogenetic analysis along with the low values of genome-based indices (dDDH and ANI) indicated that CY-1 is a member of the described *Archangium* genus. The morphology of the vegetative cell, colony, fruiting body, and predatory behavior confirmed that CY-1 had the typical characteristics of myxobacteria.

The predatory products of myxobacteria, including secondary metabolites and hydrases showing promising antimicrobial activities, have been reported (Sasse et al., 2003; Zhou et al., 2021; Zhang et al., 2023b). While most studies have focused on antibiotic production by myxobacteria, there are only a few reports about their enzymatic potential. Due to the broad prey spectrum and the complete clearing of prey biomass, myxobacteria must possess a versatile set of predation mechanisms to access nutrients from different prey species and different kinds of lytic enzymes targeting different components of prey cells. Previous studies have reported that myxobacterial glycoside hydrolases were involved in prey disintegration and showed conspicuous biocontrol efficacy in the defense of phytopathogens (Li et al., 2019; Zhang et al., 2023b). In this study, we identified a lipolytic enzyme ArEstA with bacteriolytic activity against Grame-negative drug-resistant bacteria *E. coli* 64 from the novel myxobacterium CY-1. A cold-adapted lipase with high lytic activity to *p*NP acetate from a cellulolytic myxobacterium was previously reported, but the antimicrobial activity of this enzyme was beyond the focus of their study (Cheng et al., 2011). To the best of our knowledge, our study is the first to report on the identification of lipolytic enzymes in bacteriolytic myxobacterium and its antimicrobial activity.

Myxobacteria secrete enzymes with different functions that synergistically act in the degradation of prey biomass (Berleman et al., 2014; Arend et al., 2021). We speculate that CY-1 can secrete lipases to directly degrade compounds containing ester bonds in cellular components of prey, thereby disrupting the cellular structure and achieving killing and lysis of prey. This speculation can be partially supported by the lytic activity of ArEstA to Gram-negative bacteria *E. coli* but not Gram-positive bacteria MRSA. The thick cell wall of Gram-positive bacteria prevented the access of enzymes to substrates. However, for the Gram-negative prey, the lipoprotein in the outer membrane can be easily accessed by lipase, and partial destruction of their thinner cell wall may cause access to lips in the cell membrane. Furthermore, the disruption and wrinkle of the cell envelope of *E. coli* treated with ArEstA also supported its attack on prey cells. Additionally, the combined use of exogenous lipolytic enzymes and triacylglycerols containing medium-chain fatty acids (MCFA) is a promising alternative to in-feed antibiotics in piglets due to the strong antibacterial properties of MCFA (Decuypere and Dierick, 2003). Therefore, we speculated that the bactericidal activity of ArEstA may also be related to the antibacterial activity of its hydrolysate MCFA. However, the ArEstA and glyceride reaction solution with enzyme inactivation after overnight incubation did not show bacteriolysis to *E. coli*, which indicated that the effect of MCFA for the bacteriolytic activity may be very limited in this study.

Another interesting aspect of this study is how CY-1 itself avoids the damage of the lipolytic enzyme ArEstA in predation. It is certain that the production and release of myxobacterial attacking weapons are highly regulated. Both predation and fruiting body formation of myxobacteria involve lytic action (Varon et al., 1984; Berleman et al., 2014). While predation involves the lysis of prey cells but not myxobacterial cells, autolysis of some vegetative cells provides essential requirements for the surviving cells to induce myxospores formation (Wireman and Dworkin, 1977). These different lytic actions indicate various lytic strategies and regulation systems of myxobacteria, but how they protect themselves from predation remains to be explored. The compartmentalized outer membrane vesicles packed with predatory products may help myxobacteria resistant to their own weapons (Evans et al., 2012). However, ArEstA can be expressed heterologously in *E. coli* BL21(DE3) cells but showed a bacteriolytic effect on drug-resistant *E. coli* 64 *in vitro*. We thought that the level of ArEstA background expression was very low which did not affect the growth of host cells (Pan and Malcolm, 2000), and the low protein production caused by damage of soluble ArEstA to host cells during the induction stage was overcome by expanding the amount of fermentation to obtain sufficient protein. Nevertheless, the unknown intracellular regulatory mechanism of host cells may play a role in the heterologous expression of lytic enzymes, such as proteases, lipases, and glycoside hydrolases. The bactericidal activity of ArEstA *in vitro* was independent of intracellular regulation.

## Description of *Archangium lipolyticum* sp. nov.

*Archangium lipolyticum* (li.po.ly’ti.*ca.* Gr. neut. n. *lipos*, fat; Gr. masc. adj. *lytikos*, dissolving; N.L. fem. adj. *lipolytica*, dissolving fat or lipid, referring to the property of being able to hydrolyze lipids).

Vegetative cells glide on solid media and a swarm of cells are yellow brown to purple color and film-like appearance with significant veins and flares on the periphery of the swarm. Vegetative cells are 0.5 ~ 0.6 × 5.0 ~ 12.0 μm in size with rounded poles. Sessile fruiting bodies appeared on VY/2 agar and matured to dark brown and bean-shaped. The optimal growth temperature is 30°C. The optimal pH is 7.0. Obvious growth appeared on VY/2, MD1, CTT, and CYE agar. The optimum growth temperature is 25 ~ 40°C and the optimum pH is 7.5. Skim milk, starch, and Tween 80 can be efficiently lysed. The type strain is CY-1^T^ (= GDMCC 1.3728^T^), which is isolated from a soil sample collected at a pig farm in the city of Shanwei, Guangdong Province, China. The DNA G + C content of the type strain is 68.5%, calculated from its genome sequence. The 16S rRNA gene sequence and genome sequence accession numbers of strain CY-1 in GeneBank are OR649234 and JANKBZ000000000, respectively.

## Data availability statement

The datasets presented in this study are deposited in the NCBI database under accession numbers OR649234 and JANKBZ000000000, and further inquiries can be directed to the corresponding authors.

## Author contributions

YZ: Conceptualization, Data curation, Formal analysis, Funding acquisition, Investigation, Methodology, Project administration, Software, Visualization, Writing – original draft, Writing – review & editing. HC: Formal analysis, Methodology, Data curation, Investigation, Writing – original draft. HJ: Conceptualization, Supervision, Resources, Writing – review & editing. QY: Conceptualization, Formal analysis, Resources, Supervision, Writing – review & editing. HZ: Conceptualization, Data curation, Formal analysis, Funding acquisition, Resources, Supervision, Writing – review & editing.

## Footnotes

^1^ https://www.ezbiocloud.net/tools/ani

^2^ https://services.healthtech.dtu.dk/services/SignalP-6.0/

^3^ https://www.ebi.ac.uk/Tools/msa/clustalo/

^4^ https://swissmodel.expasy.org/

## References

[ref1] Ahearne A. Phillips K. E. Knehans T. Hoing M. Dowd S. E. Stevens D. C. (2023). Chromosomal organization of biosynthetic gene clusters, including those of nine novel species, suggests plasticity of myxobacterial specialized metabolism. Front. Microbiol. 14:1227206. doi: 10.3389/fmicb.2023.1227206, PMID: 37601375 PMC10435759

[ref2] Arend K. I. Schmidt J. J. Bentler T. Lüchtefeld C. Eggerichs D. Hexamer H. M.. (2021). *Myxococcus xanthus* predation of gram-positive or gram-negative bacteria is mediated by different bacteriolytic mechanisms. Appl. Environ. Microbiol. 87:e02382-20. doi: 10.1128/AEM.02382-20 33310723 PMC8090889

[ref3] Arpigny J. L. Jaeger K. E. (1999). Bacterial lipolytic enzymes: classification and properties. Biochem. J. 343, 177–183. 10493927 PMC1220539

[ref4] Bankevich A. Nurk S. Antipov D. Gurevich A. A. Dvorkin M. Kulikov A. S.. (2012). SPAdes: a new genome assembly algorithm and its applications to single-cell sequencing. J. Comput. Biol. 19, 455–477. doi: 10.1089/cmb.2012.0021, PMID: 22506599 PMC3342519

[ref5] Berleman J. E. Allen S. Danielewicz M. A. Remis J. P. Gorur A. Cunha J.. (2014). The lethal cargo of *Myxococcus xanthus* outer membrane vesicles. Front. Microbiol. 5:474. doi: 10.3389/fmicb.2014.00474, PMID: 25250022 PMC4158809

[ref6] Berleman J. E. Kirby J. R. (2009). Deciphering the hunting strategy of a bacterial wolfpack. FEMS Microbiol. Rev. 33, 942–957. doi: 10.1111/j.1574-6976.2009.00185.x 19519767 PMC2774760

[ref7] Chambers J. Sparks N. Sydney N. Livingstone P. G. Cookson A. R. Whitworth D. E.. (2020). Comparative genomics and pan-genomics of the myxococcaceae, including a description of five novel species: *Myxococcus eversor* sp. nov., *Myxococcus llanfairpwllgwyngyllgogerychwyrndrobwllllantysiliogogogochensis* sp. nov., *Myxococcus vastator* sp. nov., *Pyxidicoccus caerfyrddinensis* sp. nov., and *Pyxidicoccus trucidator* sp. nov. Genome Biol. Evol. 12, 2289–2302. doi: 10.1093/gbe/evaa212 33022031 PMC7846144

[ref8] Cheng Y. Qian Y. Li Z. Wu Z. Liu H. Li Y. (2011). A novel cold-adapted lipase from *Sorangium cellulosum* strain So0157-2: gene cloning, expression, and enzymatic characterization. Int. J. Mol. Sci. 12, 6765–6780. doi: 10.3390/ijms12106765, PMID: 22072918 PMC3211009

[ref9] Decuypere J. A. Dierick N. A. (2003). The combined use of triacylglycerols containing medium-chain fatty acids and exogenous lipolytic enzymes as an alternative to in-feed antibiotics in piglets: concept, possibilities and limitations, an overview. Nutr. Res. Rev. 16, 193–210. doi: 10.1079/NRR200369 19087389

[ref10] DeLano W. L. Bromberg S. (2004) PyMOL user’s guide. San Carlos, CA: DeLano Scientific LLC. 629.

[ref11] Eddy S. R. (2009). A new generation of homology search tools based on probabilistic inference. Genome Inform. 23, 205–211. doi: 10.1142/9781848165632_0019, PMID: 20180275

[ref12] Evans A. G. L. Davey H. M. Cookson A. Currinn H. Cooke-Fox G. Stanczyk P. J.. (2012). Predatory activity of *Myxococcus xanthus* outer-membrane vesicles and properties of their hydrolase cargo. Microbiology 158, 2742–2752. doi: 10.1099/mic.0.060343-0 22977088

[ref13] Furusawa G. Dziewanowska K. Stone H. Settles M. Hartzell P. (2011). Global analysis of phase variation in *Myxococcus xanthus*. Mol. Microbiol. 81, 784–804. doi: 10.1111/j.1365-2958.2011.07732.x, PMID: 21722202 PMC3192537

[ref14] Garcia R. Gemperlein K. Müller R. (2014). *Minicystis rosea* gen. nov. sp. nov. a polyunsaturated fatty acid-rich and steroid-producing soil myxobacterium. Int. J. Syst. Evol. Microbiol. 64, 3733–3742. doi: 10.1099/ijs.0.068270-0 25114157

[ref1002] Goris J. Konstantinidis K. T. Klappenbach J. A. Coenye T. Vandamme P. Tiedje J. M. (2007). DNA-DNA hybridization values and their relationship to whole-genome sequence similarities. Int. J. Syst. Evol. Microbiol. 57, 81–91. doi: 10.1099/ijs.0.64483-0 17220447

[ref15] Hyatt D. Chen G. L. Locascio P. F. Land M. L. Larimer F. W. Hauser L. J. (2010). Prodigal: prokaryotic gene recognition and translation initiation site identification. BMC Bioinformatics 11:119. doi: 10.1186/1471-2105-11-119, PMID: 20211023 PMC2848648

[ref1003] Lachnik J. Ackermann B. Bohrssen A. Maass S. Diephaus C. Punken A. (2002). Rapid-cycle PCR and fluorimetry for detection of mycobacteria. J. Clin. Microbiol. 2002, 3364–3373. doi: 10.1128/JCM.40.9.3364-3373.2002 PMC130822 12202580

[ref16] Lane D. J. (1991). “16S/23S rRNA sequencing” in Nucleic acid techniques in bacterial systematics. eds. Stackebrandt E. Goodfellow M. (Chichester: Wiley), 115–148.

[ref17] Laskowski R. A. Rullmannn J. A. MacArthur M. W. Kaptein R. Thornton J. M. (1996). AQUA and PROCHECK-NMR: programs for checking the quality of protein structures solved by NMR. J. Biomol. NMR 8, 477–486. doi: 10.1007/BF00228148 9008363

[ref18] Li Z. Ye X. Chen P. Ji K. Zhou J. Wang F.. (2017). Antifungal potential of *Corallococcus* sp. strain EGB against plant pathogenic fungi. Biol. Control 110, 10–17. doi: 10.1016/j.biocontrol.2017.04.001

[ref19] Li Z. Ye X. Liu M. Xia C. Lei Z. Xue L.. (2019). A novel outer membrane β-1,6-glucanase is deployed in the predation of fungi by myxobacteria. ISME J. 13, 2223–2235. doi: 10.1038/s41396-019-0424-x, PMID: 31065029 PMC6776036

[ref20] Liu Y. Pei T. Yi S. Du J. Zhang X. Deng X.. (2021). Phylogenomic analysis substantiates the *gyrB* gene as a powerful molecular marker to efficiently differentiate the most closely related genera *Myxococcus*, *Corallococcus*, and *Pyxidicoccus*. Front. Microbiol. 12:763359. doi: 10.3389/fmicb.2021.763359, PMID: 34707598 PMC8542856

[ref21] Livingstone P. G. Ingleby O. Girdwood S. Cookson A. R. Morphew R. M. Whitworth D. E.. (2020). Predatory organisms with untapped biosynthetic potential: descriptions of novel corallococcus species *C. aberystwythensis* sp. nov., *C. carmarthensis* sp. nov., *C. exercitus* sp. nov., *C. interemptor* sp. nov., *C. llansteffanensis* sp. nov., *C. praedator* sp. nov., *C. sicarius* sp. nov., and *C. terminator* sp. nov. Appl. Environ. Microbiol. 86:19. doi: 10.1128/AEM.01931-19, PMID: 31676482 PMC6952226

[ref22] Luthy R. Bowie J. U. Eisenberg D. (1992). Assessment of protein models with three-dimensional profiles. Nature 356, 83–85. doi: 10.1038/356083a0 1538787

[ref1004] Meier-Kolthoff J. P. Auch A. F. Klenk H. Göker M. (2013). Genome sequence-based species delimitation with confidence intervals and improved distance functions. BMC Bioinformatics. 14, 1–14. doi: 10.1186/1471-2105-14-60 23432962 PMC3665452

[ref23] Meier-Kolthoff J. P. Sardà Carbasse J. Peinado-Olarte R. L. Göker M. (2022). TYGS and LPSN: a database tandem for fast and reliable genome-based classification and nomenclature of prokaryotes. Nucleic Acid Res. 50, D801–D807. doi: 10.1093/nar/gkab902, PMID: 34634793 PMC8728197

[ref24] Mohr K. I. (2018). Diversity of myxobacteria-we only see the tip of the iceberg. Microorganisms 6:84. doi: 10.3390/microorganisms6030084, PMID: 30103481 PMC6164225

[ref25] Mohr K. I. Garcia R. Gerth K. Irschik H. Müller R. (2012). *Sandaracinus mylolyticus* gen. nov. sp. nov. a starch-degrading soil myxobacterium, and description of Sandaracinaceae fam. nov. Int. J. Syst. Evol. Microbiol. 62, 1191–1198. doi: 10.1099/ijs.0.033696-0, PMID: 21742821

[ref26] Muñoz-Dorado J. Marcos-Torres F. J. García-Bravo E. Moraleda-Muñoz A. Pérez J. (2016). Myxobacteria: moving, killing, feeding, and surviving together. Front. Microbiol. 7:781. doi: 10.3389/fmicb.2016.00781, PMID: 27303375 PMC4880591

[ref27] Na S. I. Kim Y. O. Yoon S. H. Ha S. M. Baek I. Chun J. (2018). UBCG: up-to-date bacterial core gene set and pipeline for phylogenomic tree reconstruction. J. Microbiol. 56, 280–285. doi: 10.1007/s12275-018-8014-6, PMID: 29492869

[ref28] Pan S. H. Malcolm B. A. (2000). Reduced background expression and improved plasmid stability with pET vectors in BL21 (DE3). BioTechniques 29, 1234–1238. doi: 10.2144/00296st03, PMID: 11126126

[ref29] Parks D. H. Imelfort M. Skennerton C. T. Hugenholtz P. Tyson G. W. (2015). CheckM: assessing the quality of microbial genomes recovered from isolates, single cells, and metagenomes. Genome Res. 25, 1043–1055. doi: 10.1101/gr.186072.114, PMID: 25977477 PMC4484387

[ref30] Pérez J. Moraleda-Muñoz A. Marcos-Torres F. J. Muñoz-Dorado J. (2016). Bacterial predation: 75 years and counting! Environ. Microbiol. 18, 766–779. doi: 10.1111/1462-2920.13171, PMID: 26663201

[ref31] Petters S. Groß V. Söllinger A. Pichler M. Reinhard A. Bengtsson M. M.. (2021). The soil microbial food web revisited: predatory myxobacteria as keystone taxa? ISME J. 15, 2665–2675. doi: 10.1038/s41396-021-00958-2, PMID: 33746204 PMC8397742

[ref32] Richter M. Rosselló-Móra R. (2009). Shifting the genomic gold standard for the prokaryotic species definition. Proc. Natl. Acad. Sci. U. S. A. 106, 19126–19131. doi: 10.1073/pnas.0906412106, PMID: 19855009 PMC2776425

[ref33] Sasse F. Leibold T. Kunze B. Höfle G. Reichenbach H. (2003). Cyrmenins, new beta-methoxyacrylate inhibitors of the electron transport, production, isolation, physico-chemical and biological properties. J. Antibiot. 56, 827–831. doi: 10.7164/antibiotics.56.827, PMID: 14700275

[ref34] Schneiker S. Perlova O. Kaiser O. Gerth K. Alici A. Altmeyer M. O.. (2007). Complete genome sequence of the myxobacterium *Sorangium cellulosum*. Nat. Biotechnol. 25, 1281–1289. doi: 10.1038/nbt1354 17965706

[ref35] Schulz E. Goes A. Garcia R. Panter F. Koch M. Müller R. (2018). Biocompatible bacteria-derived vesicles show inherent antimicrobial activity. J. Control. Release 290, 46–55. doi: 10.1016/j.jconrel.2018.09.030, PMID: 30292423

[ref36] Seemann T. (2014). Prokka: rapid prokaryotic genome annotation. Bioinformatics 30, 2068–2069. doi: 10.1093/bioinformatics/btu153 24642063

[ref37] Shimkets L. J. Dworkin M. Reichenbach H. (2006). “The myxobacteria” in The prokaryotes. eds. Dworkin M. Falkow S. Rosenberg E. Schleifer K. H. Stackebrandt E. (New York: Springer), 31–115.

[ref38] Sievers F. Higgins D. G. (2018). Clustal omega for making accurate alignments of many protein sequences. Protein Sci. 27, 135–145. doi: 10.1002/pro.3290, PMID: 28884485 PMC5734385

[ref39] Surup F. Viehrig K. Mohr K. I. Herrmann J. Jansen R. Müller R. (2014). Disciformycins A and B: 12-membered macrolide glycoside antibiotics from the myxobacterium *Pyxidicoccus fallax* active against multiresistant staphylococci. Angew. Chem. Int. Ed. 53, 13588–13591. doi: 10.1002/anie.201406973 25294799

[ref40] Varon M. Cohen S. Rosenberg E. (1984). Autocides produced by *Myxococcus xanthus*. J. Bacteriol. 160, 1146–1150. doi: 10.1128/jb.160.3.1146-1150.1984, PMID: 6438061 PMC215832

[ref41] Waite D. W. Chuvochina M. Pelikan C. Parks D. H. Yilmaz P. Wagner M.. (2020). Proposal to reclassify the proteobacterial classes Deltaproteobacteria and Oligoflexia, and the phylum Thermodesulfobacteria into four phyla reflecting major functional capabilities. Int. J. Syst. Evol. Microbiol. 70, 5972–6016. doi: 10.1099/ijsem.0.004213 33151140

[ref42] Wang C. Lv Y. Zhou L. Zhang Y. Yao Q. Zhu H. (2022). Comparative genomics of *Myxococcus* and *Pyxidicoccus*, including the description of four novel species: *Myxococcus guangdongensis* sp. nov., *Myxococcus qinghaiensis* sp. nov., *Myxococcus dinghuensis* sp. nov., and *Pyxidicoccus xibeiensis* sp. nov. front. Microbiol. 13, 995049 13:995049. doi: 10.3389/fmicb.2022.995049, PMID: 36439860 PMC9684338

[ref43] Wang J. Wang J. Wu S. Zhang Z. Li Y. (2021). Global geographic diversity and distribution of the myxobacteria. Microbiol. Spectr. 9:e0001221. doi: 10.1128/Spectrum.00012-21 34259548 PMC8552515

[ref44] Waterhouse A. Bertoni M. Bienert S. Studer G. Tauriello G. Gumienny R.. (2018). SWISS-MODEL: homology modelling of protein structures and complexes. Nucleic Acids Res. 46, W296–W303. doi: 10.1093/nar/gky427 29788355 PMC6030848

[ref45] Wireman J. W. Dworkin M. (1977). Developmentally induced autolysis during fruiting body formation by *Myxococcus xanthus*. J. Bacteriol. 129, 798–802. doi: 10.1128/jb.129.2.798-802.1977 402359 PMC235013

[ref46] Xiao Y. Wei X. Ebright R. Wall D. (2011). Antibiotic production by myxobacteria plays a role in predation. J. Bacteriol. 193, 4626–4633. doi: 10.1128/JB.05052-11, PMID: 21764930 PMC3165673

[ref47] Ye X. Li Z. Luo X. Wang W. Li Y. Li R.. (2020). A predatory myxobacterium controls cucumber fusarium wilt by regulating the soil microbial community. Microbiome 8:49. doi: 10.1186/s40168-020-00824-x 32252828 PMC7137222

[ref48] Yoon S. H. Ha S. M. Kwon S. Lim J. Kim Y. Seo H.. (2017a). Introducing EzBioCloud: a taxonomically united database of 16S rRNA gene sequences and whole-genome assemblies. Int. J. Syst. Evol. Microbiol. 67, 1613–1617. doi: 10.1099/ijsem.0.001755 28005526 PMC5563544

[ref49] Yoon S. H. Ha S. M. Lim J. M. Kwon S. J. Chun J. (2017b). A large-scale evaluation of algorithms to calculate average nucleotide identity. Antonie Van Leeuwenhoek 110, 1281–1286. doi: 10.1007/s10482-017-0844-4, PMID: 28204908

[ref50] Zhang L. Dong C. Wang J. Liu M. Wang J. Hu J.. (2023b). Predation of oomycetes by myxobacteria via a specialized CAZyme system arising from adaptive evolution. ISME J. 17, 1089–1103. doi: 10.1038/s41396-023-01423-y 37156836 PMC10284895

[ref51] Zhang L. Huang X. Zhou J. Ju F. (2023a). Active predation, phylogenetic diversity, and global prevalence of myxobacteria in wastewater treatment plants. ISME J. 17, 671–681. doi: 10.1038/s41396-023-01378-0, PMID: 36774445 PMC9919749

[ref52] Zhao Q. Y. Li W. Cai R. M. Lu Y. W. Zhang Y. Cai P.. (2021). Mobilization of Tn1721-like structure harboring blaCTX-M-27 between P1-like bacteriophage in *Salmonella* and plasmids in *Escherichia coli* in China. Vet. Microbiol. 253:108944. doi: 10.1016/j.vetmic.2020.108944, PMID: 33370618

[ref53] Zhou Y. Yi S. Zang Y. Yao Q. Zhu H. (2021). The predatory Myxobacterium Citreicoccus inhibens gen. nov. sp. nov. showed antifungal activity and Bacteriolytic property against Phytopathogens. Microorganisms 9:2137. doi: 10.3390/microorganisms9102137, PMID: 34683458 PMC8538283

[ref54] Zhou Y. Zhang X. Yao Q. Zhu H. (2020). Both soil bacteria and soil chemical property affected the micropredator myxobacterial community: evidence from natural forest soil and greenhouse rhizosphere soil. Microorganisms 8:1387. doi: 10.3390/microorganisms8091387, PMID: 32927762 PMC7563646

